# Modulating TRPV4 Channel Activity in Pro-Inflammatory Macrophages within the 3D Tissue Analog

**DOI:** 10.3390/biomedicines12010230

**Published:** 2024-01-19

**Authors:** Parto Babaniamansour, Diego Jacho, Skyler Niedzielski, Agustin Rabino, Rafael Garcia-Mata, Eda Yildirim-Ayan

**Affiliations:** 1 Department of Bioengineering, College of Engineering, University of Toledo, Toledo, OH 43606, USA; parto.babaniamansour@rockets.utoledo.edu (P.B.); skyler.niedzielski@rockets.utoledo.edu (S.N.); 2 Department of Biological Sciences, University of Toledo, Toledo, OH 43606, USA

**Keywords:** TRPV4, macrophage, polarization, inflammation, regeneration

## Abstract

Investigating macrophage plasticity emerges as a promising strategy for promoting tissue regeneration and can be exploited by regulating the transient receptor potential vanilloid 4 (TRPV4) channel. The TRPV4 channel responds to various stimuli including mechanical, chemical, and selective pharmacological compounds. It is well documented that treating cells such as epithelial cells and fibroblasts with a TRPV4 agonist enhances the Ca^2+^ influx to the cells, which leads to secretion of pro-inflammatory cytokines, while a TRPV4 antagonist reduces both Ca^2+^ influx and pro-inflammatory cytokine secretion. In this work, we investigated the effect of selective TRPV4 modulator compounds on U937-differentiated macrophages encapsulated within three-dimensional (3D) matrices. Despite offering a more physiologically relevant model than 2D cultures, pharmacological treatment of macrophages within 3D collagen matrices is largely overlooked in the literature. In this study, pro-inflammatory macrophages were treated with an agonist, 500 nM of GSK1016790A (TRPV4(+)), and an antagonist, 10 mM of RN-1734 (TRPV4(−)), to elucidate the modulation of the TRPV4 channel at both cellular and extracellular levels. To evaluate macrophage phenotypic alterations within 3D collagen matrices following TRPV4 modulator treatment, we employed structural techniques (SEM, Masson’s trichrome, and collagen hybridizing peptide (CHP) staining), quantitative morphological measures for phenotypic assessment, and genotypic methods such as quantitative real-time PCR (qRT-PCR) and immunohistochemistry (IHC). Our data reveal that pharmacological modulation of the macrophage TRPV4 channel alters the cytoskeletal structure of macrophages and influences the 3D structure encapsulating them. Moreover, we proved that treating macrophages with a TRPV4 agonist and antagonist enhances the expression of pro- and anti-inflammatory genes, respectively, leading to the upregulation of surface markers CD80 and CD206. In the TRPV4(−) group, the CD206 gene and CD206 surface marker were significantly upregulated by 9- and 2.5-fold, respectively, compared to the control group. These findings demonstrate that TRPV4 modulation can be utilized to shift macrophage phenotype within the 3D matrix toward a desired state. This is an innovative approach to addressing inflammation in musculoskeletal tissues.

## 1. Introduction

Transient receptor potential vanilloid 4 is a nonselective Ca^2+^-permeable cation channel that belongs to the transient receptor potential (TRP) subfamily [1,2]. The TRPV4 channel exhibits membranous expression and can be found in the brain, eyes, urinary system, gastrointestinal tract, skin, epithelial, vasculature, several musculoskeletal tissues, and innate immune system cells [2,3]. The TRPV4 ion channel activity is affected by changes in the cell membrane following heat, swelling, pH, mechanical, and biological stimuli [1,4]. Without these external stimuli, the TRPV4 activity can be blocked or enhanced in a controllable fashion using selective pharmacological compounds including selective antagonists, RN-1734, HC-067047, GSK2193874, and GSK2798745, and selective agonists 4aPDD, RN-1747, and GSK1016790A [5]. The TRPV4 antagonists bind to the cell membrane creating conformational changes in the pore region of the TRPV4 channel and inhibit the calcium uptake [6]. The TRPV4 channel agonists expand the plasma membrane and widen the pore-forming region of TRPV4 which subsequently enhances Ca^2+^ influx into the cells, triggering the down-signaling pathways, and translating into changes in gene expression [7,8].

TRPV4 channel de/activation affects inflammation through several distinct mechanisms: (i) TRPV4 channel activation modulates inflammation by recruiting immune cells such as macrophages to the local inflammation site [9,10,11]. For example, Sumioka et al. [12] showed decreased infiltration of macrophages to the injury site of TRPV4−/− mice [12]. (ii) TRPV4 channel activation induces calcineurin activation in many cells, leading to increased expression of nuclear factor of activated T-cells (NFAT) and nuclear factor kappa B (NF-κB) [13]. For example, Li et al. [14] found that GSK1016790A treatment in mouse retinal glial cells upregulated NF-κB expression [14]. (iii) TRPV4 channel activation enhances the release of inflammatory cytokines, chemokines, and nitric oxide (NO) in various cell types, including epithelial cells, fibroblasts, and immune cells [13,15]. For example, Henry et al. [16] showed that TRPV4 agonist increased Ca^2+^ influx in human respiratory epithelial cells which further enhanced PGE2 production and IL-8 secretion [16]. Conversely, blocking the TRPV4 channel reduced Ca^2+^ entry and lowered the level of the proinflammatory cytokine [17,18,19]. Similarly, H. Shannon et al. [20] showed that mice receiving a daily intraperitoneal injection of GSK2193874 demonstrated reduced inflammatory cytokine IL-1β and inflammatory chemokine MIP2 [20]. (iv) TRPV4 channel activation shifts macrophages, the key regulators of inflammation, toward more pro-inflammatory phenotypes. For example, Hamanaka et al. [21] showed increased levels of reactive oxygen species (ROS) and reactive nitrogen species (RNS) in alveolar macrophages from TRPV4+/+ mice [21]. Conversely, in macrophages treated with a TRPV4 antagonist, the pro-inflammatory properties are attenuated. For example, Sun et al. [22] showed that intra-articular injection of HC067047 reduced M1 marker expression (IL-1β, IL-6, and iNOS) and alleviated the osteoarthritis (OA) progression in rats by decreasing synovial M1 macrophage polarization [22]. Similarly, Pairet et al. [23] showed that treating M1 macrophages seeded on the plate with TRPV4 antagonist abolished the secretion of pro-inflammatory cytokines such as IL-1β, TNF-α and IL-6.

All of these studies shed light on the importance of TRPV4 channels for hemostasis and inflammation. Yet, so far, these studies and many others in the literature characterize the macrophage phenotypic changes following TRPV4 modulation through assessing the changes in the cytokine from the media or supernatant using multiplex cytokine assay kits because of their efficiency, high throughput capabilities, and use of smaller sample volumes. However, the multiplex cytokine assay solely measures cytokines in the media or supernatant, which omits the crucial information and context of the three-dimensional (3D) tissue where the cells are residing. Consequently, information regarding tissue–cell interactions, cytokine production within the tissue, and the influence of the tissue microenvironment on cytokine production is lost. Furthermore, multiplex assays do not offer spatial information on cytokine production within the tissue, and the potential for dilution effects and artifacts from cell culturing can limit their accuracy. Researchers often complement multiplex assays with other techniques, such as immunohistochemistry or in vivo studies, to gain a more comprehensive understanding of cytokine responses within 3D tissue environments. Furthermore, these investigations have primarily been conducted on macrophages seeded on two-dimensional (2D) cell culture plates (Petri dishes and flasks) or with in vivo models. While working with 2D in vitro studies using cost-effective and straightforward protocols, the macrophages on 2D surfaces display different cellular functions and polarization potential compared to the counterparts encapsulated within the 3D matrices [24]. The spatial macrophage confinement within the 3D matrix influences the plasma membrane’s lipid bilayer, further creating tension-dependent energy differences promoting TRPV4 ion channel conformational changes and activation. Moreover, when physical environments hinder cell spreading, the total histone deacetylase 3 (HDAC3) decreases, which attenuates the macrophage differentiation toward the pro-inflammatory phenotype [24]. These findings emphasize the importance of studying the effects of TRPV4 modulators on 3D-encapsulated macrophage phenotypes instead of relying on traditional 2D systems.

The influence of TRPV4 modulators on macrophage polarization is commonly studied using the in vivo models, which provide superior physiological relevance compared to 2D and 3D in vitro studies [25,26,27]. However, with in vivo models, the systemic administration of TRPV4 modulators throughout the body potentially leads to off-target effects since TRPV4 channels are present on much of the mammalian cell membrane. In addition, using in vivo models, it is challenging to isolate the solo effect of TRPV4 in macrophage phenotypic commitment. Thus, there is a great demand for a comprehensive study elucidating the macrophages’ phenotypic transition within a 3D matrix through analyzing cell–tissue interaction, and intracellular and extracellular changes in response to TRPV4 modulators.

Toward this end, the objective of this study is to understand how modulating macrophage TRPV4 activation within the in vitro 3D matrix affects macrophage phenotypic commitment and macrophage–matrix interaction characterized by comprehensive analysis including gene-, protein-, and structural-level characterizations. We have used reverse transcription-polymerase chain reaction (RT-PCR) to study the relative fold change in the expression of pro- and anti-inflammatory genes along with immunohistochemistry staining targeting CD80 and CD206 to ascertain the expression of surface markers. Additionally, Masson’s trichrome staining and collagen hybridizing peptide (CHP) staining were performed on 3D matrices to study collagen density alteration in the macrophage microenvironment. The result of this study can shed light on immunomodulatory strategies that focus on inhibiting the TRPV4 channel to resolve inflammation and enhance tissue regeneration.

## 2. Materials and Methods

### 2.1. Human Macrophage Cultivation

The human pro-monocytic cell U937 (ATCC, Manassas, VA, USA) was cultured in Roswell Park Memorial Institute (RPMI) 1640 medium (ATCC, Manassas, VA, USA), supplemented with 10% (*v*/*v*) heat-inactivated fetal bovine serum (FBS; ThermoFisher, Waltham, MA, USA), 2 mM L-glutamine, 10 mM HEPES, 1 mM sodium pyruvate, 4500 mg/L glucose, and 1500 mg/L sodium bicarbonate and kept in the incubator (37 °C, 5% CO_2_) for expansion. After changing the medium every 2–3 days and upon reaching the confluency, the U937 monocyte cells were treated with 100 ng/mL of phorbol 12-myristate 13-acetate (PMA) (Sigma-Aldrich, St. Louis, MO, USA) for 24 h to be differentiated into naïve macrophages (M0). To polarize M0 macrophages to pro-inflammatory (M1) phenotype, the cells were further cultured within the media supplemented with 20 ng/mL interferon-gamma (IFNγ, Peprotech, Cranbury, NJ, USA) and 100 ng/mL lipopolysaccharide (LPS) (Sigma, Ronkonkoma, NY, USA) for 24 h based on our validated protocol [28].

### 2.2. Treatment of Macrophages with TRPV4 Ion Channel Modulators

The GSK1016790A (Millipore Sigma, Burlington, MA, USA, G0798) and RN-1734 (Millipore Sigma, R0658) were used as an agonist and an antagonist for TRPV4 ion channels, respectively. These modulators were chosen based on their level of selectivity [29,30]. For activating TRPV4 channels, GSK1016790A was added to the macrophage media with a concentration of 500 nM. The concentration was chosen based on published literature data based on cell viability and efficiency [31,32]. For blocking TRPV4 channels, RN-1734 was added into the media with 10 µM concentration following concentration optimization analysis. Briefly, to identify the optimal concentration for the TRPV4 antagonist, the macrophages were cultured with 10 nM, 1 mM, and 10 µM RN-1734, and cell viability was measured using a Live-Dead Assay kit (Life Technologies, Carlsbad, CA, USA). Following incubating macrophages for 48 h with various antagonist concentrations, the cells were washed with dPBS and cultured with 2 mM of Calcein AM and 4 mM ethidium homodimer-1 dyes for 2.5 h at 37 °C and then fixed with 4% paraformaldehyde (Sigma, USA) for 30 min. The live and dead cells were then visualized using fluorescence microscopy at 490/525 nm and 557/576 nm excitation/emission wavelengths to visualize live (green) and dead (red) cells, respectively.

### 2.3. Confirmation of Activation/Deactivation of TRPV4 Channels upon TRPV4 Modulators using TRPV4 Antibody Staining and Intracellular Calcium Level

To confirm the effectiveness of GSK1016790A and RN-1734 as a TRPV4 channel agonist and antagonist, the macrophages were incubated with either 500 nM GSK1016790A or 10 µM RN-1734 for 24 h and evaluated by immunofluorescence staining and level of intracellular calcium. The macrophages cultured without a TRPV4 modulator in media were used as a control.

For immunofluorescence staining (IF), following incubation with TRPV4 modulators or media, the cells were fixed with 4% paraformaldehyde (Sigma, USA) for 15 min and were washed with PBS three times. The cells were then quenched with ammonium chloride for 15 min followed by three washes with PBS. The cells were permeabilized with 0.1% Triton x-100 in PBS and washed again with PBS. The cells were then blocked in 2.5% goat serum (Invitrogen, Waltham, MA, USA), and 0.2% Tween in PBS for 20 min. They were washed and incubated overnight at 4 °C in an anti-TRPV4 antibody (Millipore Sigma, MABS466). The next day, the cells were washed with PBS, and 0.2% Tween solution and blocked in 0.4% BSA, and 0.2% Tween solution for 20 min at room temperature (RT). After blocking, the cells were incubated for 2 h at RT with Alexa fluor 488-Goat anti-mouse IgG (A32730, ThermoFisher, USA) (1:50) and were washed with 0.2% Tween solution. Finally, the cells were imaged using OLYMPUS IX71 confocal microscopy at 490/520 nm. The acquired images were then post-processed and analyzed using ImageJ. Briefly, fluorescent images from at least three fields of view in each treatment group were analyzed, and the intensity of the anti-TRPV4 antibody in 100 cells per group was measured. The integrated density of each cell was obtained and then divided by the cell area to signal intensity per unit area for each cell. For normalization, the background integrated density value was measured and then subtracted from the pixel values of the cell area.

**Intracellular calcium level assessment:** The macrophages were seeded in a 96-well plate with a density of 50K cells/well and incubated with PBS solution containing 5 μM of Fura-2 AM (F1222, ThermoFisher Scientific, USA) and 0.1% *v*/*v* Pluronic™ F-127 for 2 h at 37 °C. Then, cells were washed with PBS to remove excess Fura-2 dye and treated with 500 nM agonist and 10 μM of TRPV4 antagonist. Real-time fluorescent calcium signals were recorded using a SpectraMax M2 microplate reader equipped with SoftMax Pro 7.1 software for 20 min. The fluorescent value was obtained by exciting the cells at two wavelengths (340 nm and 380 nm) with an emission wavelength of 510 nm. The ratio of fluorescence intensity in agonist (TRPV4(+)) and antagonist (TRPV4(−)) macrophages at the two excitation wavelengths (340/380) was calculated and compared to naive macrophages (control group) to evaluate the impact of TRPV4 agonist/antagonist on intracellular calcium dynamics.

### 2.4. Synthesizing Macrophage-Laden 3D Tissue Matrix

**Figure 1:**
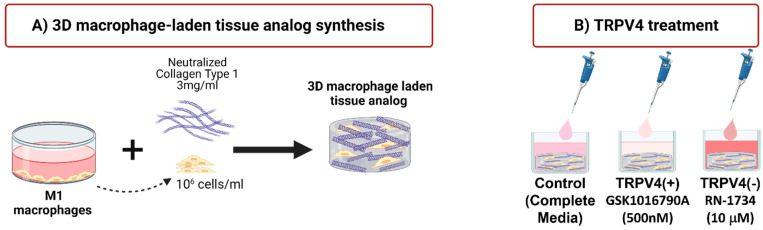
Schematic representations of: (**A**) synthesizing macrophage-laden 3D tissue matrix, and (**B**) supplementation with complete media (control), TRPV4 agonist, and TRPV4 antagonist.

To create a 3D M1-laden tissue matrix, the M1 macrophages were (encapsulated with the neutralized 3 mg/mL collagen type-1 (Ibidi, Fitchburg, WI, USA) with a seeding density of 1 × 10^6^ cells/mL following our established protocols [28,33,34]. Collagen type-I was chosen as a tissue matrix material due to its prevalence in the extracellular matrix (ECM) of most musculoskeletal tissues [35]. Then, 750 mL of macrophage-laden 3D tissue matrices was dispensed into the well plate and incubated for 2 h to allow for collagen polymerization. Following 2 h incubation, the macrophage-laden 3D tissue matrices were divided into three groups of control, TRPV4(+), and TRPV4(−) which were supplemented with complete RPMI-1640, 500 nM of GSK1016790A, and 10 mM of RN-1734, respectively. Figure 1 demonstrates the significant steps for cell cultivation and macrophage-laden tissue matrix synthesis.

### 2.5. Structural Changes in 3D M1-Laden Tissue Matrix upon TRPV4 Modulators

Structural changes in pharmacologically activated M1 macrophages encapsulated by the 3D matrix were assessed using Masson’s trichrome and B-Collagen hybridizing peptide (CHP)-stained images. After 4 days of incubation with TRPV4 modulators (GSK1016790A and RN-1734), macrophage-laden tissue matrices were fixed in 10% formalin overnight, dehydrated in graded ethanol, and embedded in paraffin. The tissue matrices were sliced with a thickness of 5 μm using a microtome (GMI-Reichert Jung 820 II) and mounted on a glass microscopic slide (Mercedes Medical MER 7200/45/BL) to be stained with Masson’s trichrome for visualization of collagen deposition.

Moreover, to visualize and quantify collagen degradation in the matrices, 8 μm thickness slices were provided, mounted on the microscopic slide, and kept in the oven overnight at 55 °C. The B-CHP staining, which tags the degraded and proteolyzed collagen matrix, agreed with the histological data. The B-CHP staining was performed per the manufacturer’s protocol (50 μM; B-CHP #BIO-300; 3Helix, Salt Lake City, UT, USA) [36]. Briefly, B-CHP was diluted in PBS (20 μM) and heated to 80 °C for 5 min to avoid self-assembly of CHP into triple helices. Then, diluted B-CHP was quickly cooled in ice for 1 min to avoid thermal damage to the samples. Samples were incubated with B-CHP (20 μM) at 4 °C overnight and washed with PBS for 5 min at room temperature. Then, slides were incubated in 0.005 mg/mL of Alexa Fluor 488 streptavidin conjugate in a PBS solution containing 1% BSA for 2 h at room temperature. After washing the slides with PBS for 5 min, they were imaged using OLYMPUS IX71 and the cellSens Dimension software (Version 2022). Samples were then post-processed and analyzed using ImageJ software (Version 2023). Briefly, fluorescent images from five fields of view from each treatment group were processed to measure fluorescent integrated density per area. The integrated density values were corrected by subtracting the background integrated density values and dividing by the value area to determine the CHP fluorescent intensity/unit area (CHP signal) for each treatment group. It was of note that the autofluorescence signal was negligible with the chosen imaging settings.

### 2.6. Identifying the Effect of TRPV4 Modulators on Macrophage Phenotypic Profile

**Gene expression analysis:** To study the effect of TRPV4 modulators on phenotypic changes in macrophages, quantitative real-time polymerase chain reaction (qRT-PCR) was performed. The macrophages were incubated with complete media or TRPV4 antagonist or TRPV4 agonist with aforementioned TRPV4 modulator concentrations. Then, RNA from each group was extracted using TRIzol reagent (ThermoFisher Scientific, USA). The isolated RNA was reverse transcribed using the Omniscript RT kit (Qiagen, Germantown, MD, USA) per the manufacturer’s instructions. The qRT-PCR was performed using the SYBR Green PCR master mix (ThermoFisher Scientific, USA) for detecting the expression of pro- and anti-inflammatory genes. The relative fold changes between TRPV4(+), TRPV4(−), and control (no reagent) were obtained using the ∆∆Ct method with glyceraldehyde-3-phosphate dehydrogenase (GAPDH) serving as the housekeeping gene for normalization. The qRT-PCR experiments were conducted using the iCycler iQ detection system (Bio-Rad, Hercules, CA, USA) with a total of 35 thermocycling cycles. Primer sequences were obtained from published literature, as listed in Table S1 (Supplementary Material), and Integrated DNA Technologies (IDT, Arlington, VA, USA). Subsequently, relative fold changes of pro- and anti-inflammatory genes were further analyzed and visualized using open-source software (http://www.heatmapper.ca (accessed on 15 September 2023)). The heatmaps were constructed using the average linking method in conjunction with the Euclidean method. The heatmap allowed for a comparison of gene expression related to the activation and inhibition of the TRPV4 ion channel in M1 macrophages.

**Cell Surface Markers Analysis (Immunostaining):** Following 4 days of incubating 3D M1-laden matrices with TRPV4 modulators, the expression of pro- and anti-inflammatory markers was confirmed using fluorescent staining. Briefly, scaffolds were fixed in 10% formalin, dehydrated in graded ethanol, and embedded in paraffin. The tissue matrices were sliced with a thickness of 40 μm using a microtome (GMI-Reichert Jung 820 II), mounted on a glass microscopic slide (Mercedes Medical MER 7200/45/BL) and kept in the oven overnight at 80 °C. The next day, slides were immersed in xylene for 2 h to remove the remaining paraffin. The slides were then transferred to 100%, 95%, 70%, and 50% ethanol solution, respectively. Slides were rinsed in diH_2_O. Following deparaffinization and rehydration, the slides were incubated in trypsin and calcium chloride solution (0.1% (*v*/*v*) trypsin (ATCC, USA) plus 0.1% (*w*/*v*) calcium chloride (Sigma-Aldrich, USA)) at 37°C for 30 min. Then, heat-mediated antigen retrieval was performed by immersing the slides in citrate buffer (10 mM sodium citrate in 0.05% Tween 20) at 95 °C for 30 min. Then, samples were permeabilized with Triton x-100 for 10 min, washed in PBS, and incubated with 2.5% (*v*/*v*) goat serum (Invitrogen) and 0.2% Tween solution for 20 min to block non-specific antibody binding. Slides were subsequently incubated overnight at 4 °C in mouse monoclonal CD80 antibody (1:100) (Invitrogen, TA501575) and recombinant rabbit CD206 antibody (1:100) (ThermoFisher, MA1-35936). CD80 and CD206 were chosen because they are pro-inflammatory and anti-inflammatory macrophage-related surface markers, respectively [37]. The next day, slides were washed with PBS and 0.2% Tween solution and blocked in 2.5% (*v*/*v*) goat serum (Invitrogen) and 0.2% Tween solution for 20 min at room temperature. Then, slides were incubated with Alexa Flour 488 Phalloidin (1:200) (ThermoFisher, A12379), goat anti-rabbit IgG (H+L) Highly Cross-Adsorbed Secondary Antibody Alexa Fluor 594 (1:200) (ThermoFisher, A32740) and goat anti-mouse IgG (H+L) Highly Cross-Adsorbed Secondary Antibody, Alexa Fluor 647 (ThermoFisher, A32728TR) for 4 h and 30 min. Finally, slides were incubated with DAPI (4′,6-Diamidino-2-Phenylindole) (Life Technologies) dye (1:1000) in PBS for 30 min and washed with 0.2% Tween. Slides were imaged using a 63X oil or 20X dry objective with a Leica Stellaris 5 confocal system equipped with HyD detectors and the LASX software. Confocal images from at least three independent studies were processed to visualize each cell’s nuclei, filamentous actin (F-actin), and CD80 and CD206 expressions. To quantify pro- and anti-inflammatory marker expression, confocal images of TRPV4(+), TRPV4(−), and control macrophage samples were post-processed and analyzed with ImageJ software (version 2023) to measure a fluorescent signal associated with Alexa Fluor 647 and Alexa Fluor 594 secondary antibody. The integrated density values obtained for each cell were corrected by subtracting the background signal and normalized by dividing by cell surface area.

### 2.7. Statistical Analysis

Six samples (n = 6) were used for all assays, and the statistical analysis was performed through RStudio. Statistical significance was conducted using one-way analysis of variance (ANOVA) and post-hoc analysis (Tukey test) or Student’s *t*-test where appropriate. The (*) represents a significant difference between the groups with a 0.05 *p*-value. A double symbol (**) shows that the significance level in statistical analysis between the groups is *p* < 0.005 instead of *p* < 0.05. In graphs, the error bars represent the standard deviations unless otherwise specified. 

## 3. Results

### 3.1. Validating the Effectiveness of TRPV4 Agonist and Antagonist on Macrophages

Prior to studying in detail how the polarization of macrophages within the 3D matrix changes with the pharmaceutical modulation of TRPV4 ion channels, the effectiveness of TRPV4 agonists and antagonists on naïve macrophages was validated using comprehensive analyses including cell viability, changes in intracellular Ca^2+^ upon treatment, TRPV4 expression in protein level upon treatment, and inflammatory gene expressions.

**Figure 2:**
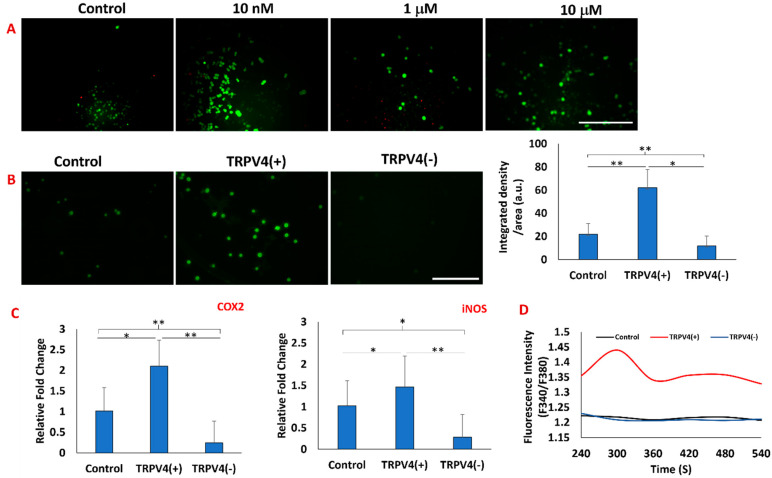
(**A**) Double staining of live (green) and dead (red) macrophages treated with various concentrations (10 nM, 1 mM, and 10 mM) of RN-1734. The scale bar represents 500 μm. (**B**) Immunofluorescence (IF) staining of macrophages treated with TRPV4 antagonist (RN-1734) and agonist (GSK1016790A) and fluorescence intensity quantification. n = 100 cells were used for analysis. The scale bar represents 50 μm. (**C**) Effect of TRPV4 modulators on the expression of COX2 and iNOS. * Indicates a significant difference between the control group and the treated group with *p* < 0.05. ** Indicates a significant difference between the control group and the treated group with *p* < 0.005. (**D**) Effect of TRPV4 modulators on calcium influx into macrophages.

First, the effect of different concentrations (10 nM, 1 mM, and 10 mM) of TRPV4 antagonist RN-1734 on the viability of macrophages within the 3D matrix was assessed by live/dead double staining (Figure 2A). The live/dead images demonstrated that even the highest RN-1734 concentration (10 µM) did not affect the cell viability. Thus, 10 µM RN-1734 was utilized for the rest of the study as a TRPV4 antagonist concentration. Then, upon treatment with TRPV4 antagonist and agonist, the TRPV4 ion channel expression was assessed for macrophages within the 3D matrix using immunohistochemistry. The results of immunofluorescence staining with anti-TRPV4 antibody and image analysis (Figure 2B) revealed that the TRPV4 agonist, GSK1016790A, significantly increased the expression of TRPV4 channels on the macrophages. The TRPV4 agonist treatment increased the fluorescent intensity of TRPV4 channel expression on cells 3-fold compared to the control group. As expected, the TRPV4 antagonist-treated group, TRPV4(−), had the lowest fluorescent intensity among the counterparts in the control and agonist treatment groups.

It has already been shown that activation of the TRPV4 channel enhances inflammatory response in the temporomandibular joint (TMJ), disc cells, hippocampal cells, and alveolar macrophages [15,38,39]. Thus, to ensure that TRPV4 antagonists and agonists effectively work on macrophages within the 3D matrix, the expression of COX2 and iNOS genes was assessed following 24 h treatment using qRT-PCR. The gene expression data (Figure 2C) demonstrated that TRPV4 agonist treatment increased the expression of inflammatory markers of COX2 and iNOS compared to control and TRPV4 antagonist treatment groups. As illustrated in Figure 2C, the COX2 and iNOS relative fold-changes in macrophages treated with RN-1734 showed a 5-fold decrease compared to control whereas macrophages treated with GSK1016790A exhibited a 2-fold increase. The iNOS relative fold-change in macrophages treated with GSK1016790A showed a 1.5-fold increase compared to the control group.

Finally, to determine whether the pharmacological modulation of the TRPV4 channel mediates Ca^2+^ influx, intracellular calcium was measured using the Fura-2 assay. The calcium influx data (Figure 2D) demonstrated that upon TRPV4 agonist treatment, the calcium influx rapidly increased within 300 s and then stabilized. For macrophages in the TRPV4(−) group and in the control group (no treatment), the intracellular calcium was 20% lower compared to the TRPV4(+) group. As a result, higher (F_340_/F_380_) observed in macrophages treated with TRPV4 agonist confirms Ca^2+^ influx associated with overexpression and activation of TRPV4 channel in macrophages.

### 3.2. The Effect of Pharmacological Activation of TRPV4 Channel on Collagen Matrix Surrounding the Macrophages

The M1 macrophages encapsulated within the 3D collagen type-I matrix were treated with a validated agonist and antagonist to understand the effect of pharmacological activation of the TRPV4 channel on the collagen matrix circulating the macrophages. The structural changes of M1-laden 3D collagen matrix upon TRPV4 agonist and TRPV4 antagonist treatment were studied by assessing the structural integrity of collagen matrix and degraded collagen using histological analysis (Masson’s trichrome) and fluorescence imaging (B-CHP images).

**Figure 3:**
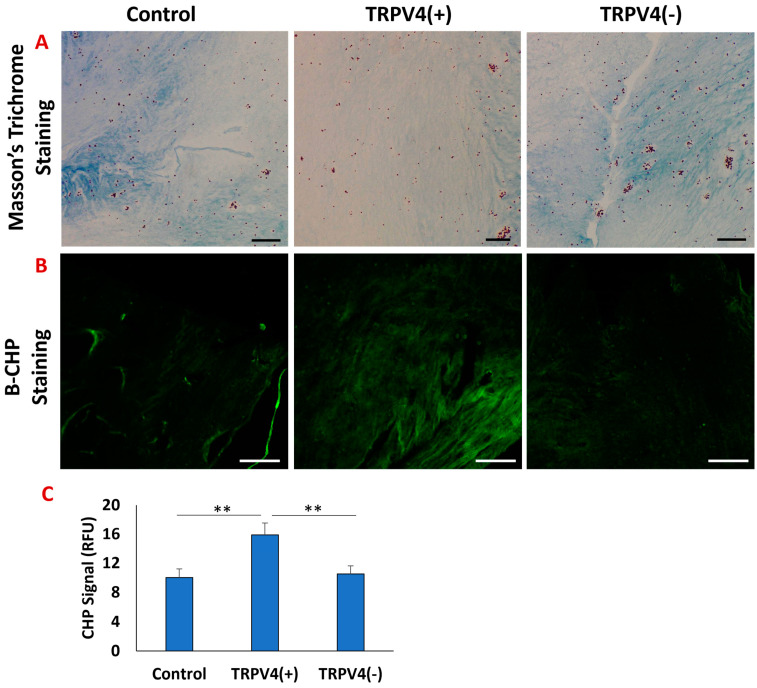
The histological images (**A**) and B-CHP stained fluorescence images (**B**) of the 3D collagen matrix encapsulated M1 (Control), TRPV4(+), and TRPV4(−) macrophages, respectively. The scale bar represents 200 mm for both (**A**,**B**). (**C**) The bar graph represents the mean fluorescent intensity. ** Indicates significant difference between two different groups, with a *p* < 0.005. Five random fields of view within the histological images were used for quantification.

Figure 3A shows the Masson’s trichrome staining and Figure 3B shows the B-CHP staining of the 3D collagen matrix with M1 macrophages residing in it under TRPV4 agonist treatment (TRPV4(+)), TRPV4 antagonist treatment (TRPV4(−)), and no treatment (control). In Masson’s trichrome staining, the collagen fibers stained in blue while the cell nucleus stained with purple color. The collagen matrix in TRPV4(+) group demonstrated a less dense collagen network compared to the control and TRPV4(−) groups. This result suggests that the pharmacological activation of the TRPV4 channel in M1 macrophages decreased the overall structural integrity of the collagen matrix. The collagen density in the TRPV4(−) and control groups were similar, with a denser structure than their TRPV4(+) counterparts. Figure 3B shows the B-CHP staining of the M1-laden 3D collagen matrix and Figure 3C demonstrates the changes in B-CHP fluorescent signals upon TRPV4(+) and TRPV4(−) treatment and control groups. The degraded collagen images (Figure 3B) demonstrated that the M1-laden collagen matrix treated with TRPV4 agonist (TRPV4(+)) has undergone more collagen degradation as a higher amount of degraded collagen matrix was tagged with B-CHP compared to TRPV4(−) and control groups. As expected, the highest fluorescent intensity was also observed in TRPV4(+) group with 15.87 ± 1.62 RFU, while the fluorescent intensities of B-CHP in TRPV4(−) and control groups were 10.04 ± 1.15 and 10.53 ± 1.11 RFU, respectively. There was no statistical difference between the B-CHP signals of matrices treated with TRPV4(−) and control groups.

### 3.3. The Effect of Pharmacological Activation of TRPV4 Channels on the Cytoskeletal Structure of Macrophages within the 3D Collagen Matrix

**Figure 4:**
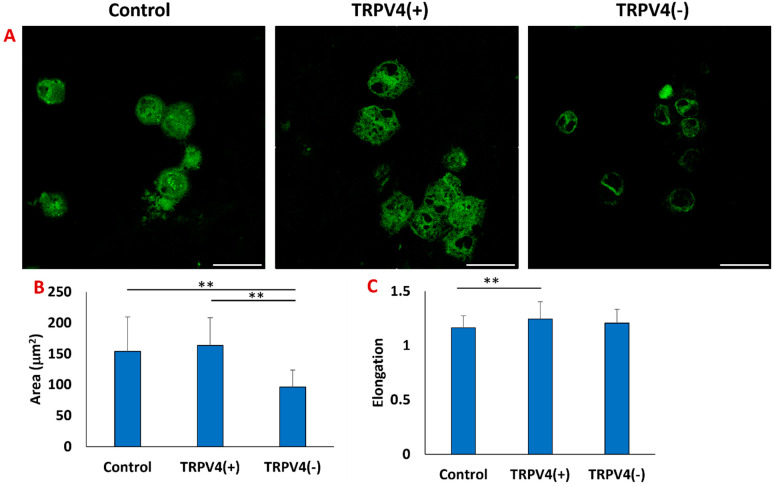
(**A**) Representative confocal image of the M1 (Control), TRPV4(+), and TRPV4(−) within a 3D matrix stained with Alexa Fluor 488 phalloidin. The scale bar represents 20 μm. (**B**) Bar graph represents the quantification of the surface area of each treatment group. n = 50 cells were used in image analysis. (**C**) Bar graph represents the quantification of cell elongation of each treatment group. n = 50 cells were used in image analysis. ** shows that the statistical difference has a significance level of *p* < 0.005.

After we confirmed the structural changes of the M1-laden 3D collagen matrix upon pharmacological activation of the TRPV4 channel, we studied whether this mechanical strain is translated to cytoskeletal changes in collagen-embedded macrophages. The intracellular filamentous actin (F-actin) geometry was assessed in TRPV4(+), TRPV4(−), and the control group (Figure 4).

The morphological characteristics of the macrophages, such as surface area and elongation, vary among distinct polarization states [40]. Previous studies indicate that macrophages respond to soluble factors like LPS and IL-4 with morphological changes [41]. Our findings using F-actin immunofluorescent staining reveal that pharmacological modulation of the TRPV4 channel also alters macrophage morphology. Figure 4A illustrates that both the control and TRPV4(+) groups display larger surface areas, measuring 153.87 ± 55.41 mm^2^ and 163.46 ± 44.82 mm^2^, respectively. However, these dimensions are statistically larger when compared to the size of the TRPV4(−) group, which is 96.04 ± 27.77 mm^2^. Moreover, there was a statistically significant increase in cellular elongation in the TRPV4(+) group compared to the control and TRPV4(−) groups. The cell elongation, quantified as the ratio of length to width, exhibited the values of 1.16 ± 0.11, 1.24 ± 0.15, and 1.20 ± 0.12 for control, TRPV4(+), and TRPV4(−) groups, respectively.

### 3.4. The Effect of Pharmacological Activation of TRPV4 Channel on Phenotypic Changes of M1 Macrophages within the 3D Collagen Matrix

**Figure 5:**
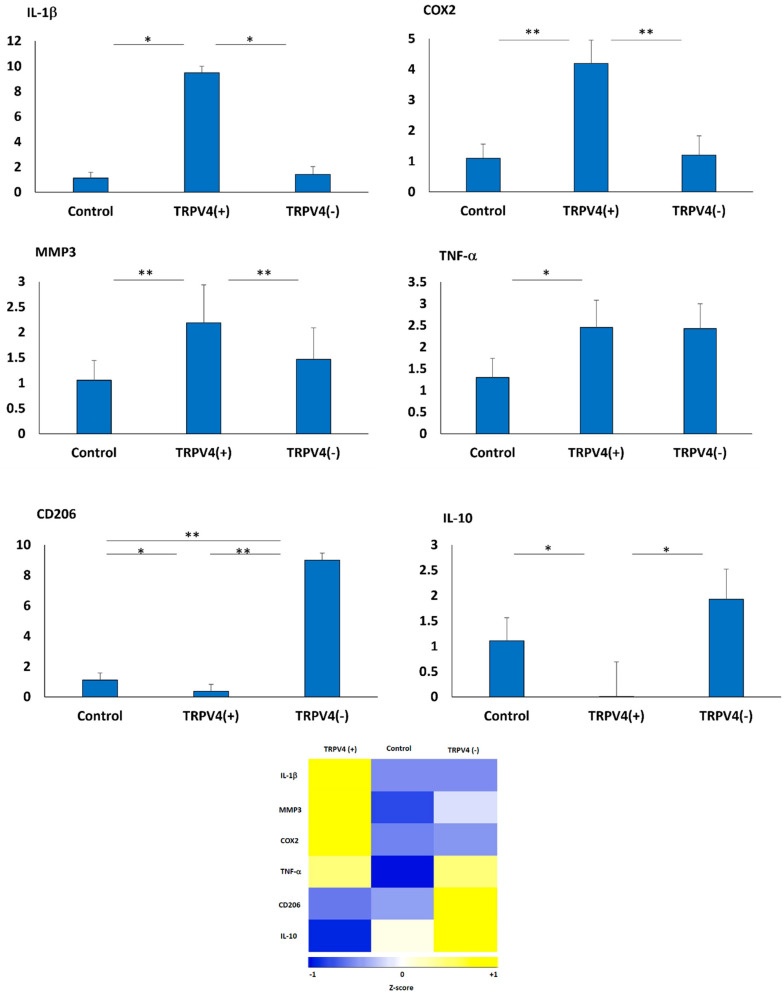
Effect of TRPV4 agonist (TRPV4(+)) and antagonist (TRPV4(−)) treatment on the gene expression of pro-inflammatory, matrix degradation markers, and anti-inflammatory genes in M1-laden 3D collagen matrix. * Shows that the statistical difference has a significance level of *p* < 0.05 and ** shows that the statistical difference has a significance level of *p* < 0.005. The heatmap of pro- and anti-inflammatory gene expressions changes in response to treatment with TRPV4 modulators. The yellow indicates gene upregulation while the blue color shows gene downregulation.

After treating M1-laden 3D collagen matrices with GSK1016790A and RN-1734 for 4 days, we have investigated whether applying TRPV4 activating/blocking agent modulates the phenotypic changes of pro-inflammatory macrophages within the 3D collagen matrix. The changes in relative expressions of prominent pro- and anti-inflammatory markers along with matrix degradation markers were assessed using qRT-PCR. The relative fold-change in expressions of pro- and anti-inflammatory genes including TNF-α, IL-1β, COX2, CD206, IL-10, and MMP3 for TRPV4(+) and TRPV4(−) groups and control (no treatment) group are given in Figure 5.

For the TRPV4(+) group, all pro-inflammatory genes (IL-1β, COX2, and TNF-α), as well as the matrix degradation marker MMP3, were significantly (*p* < 0.05) upregulated, while the anti-inflammatory genes IL-10 and CD206 were downregulated compared to their counterparts in the control group (no treatment). As Figure 5 shows, in the TRPV4(+) macrophages encapsulated within 3D matrix, IL-1β, COX2, MMP3 and TNF-α were significantly upregulated with 9.47 ± 0.51-fold, 4.18 ± 0.76-fold, 2.18 ± 0.75-fold, and 2.45 ± 0.62-fold compared to control. However, the relative fold change of CD206 and IL-10 in TRPV4(+) macrophages downregulated significantly with 0.37 ± 0.45-fold, and 0.009 ± 0.68-fold, respectively.

For the TRPV4(−) group, the anti-inflammatory genes (IL-10 and CD206) were significantly upregulated compared to their counterparts in TRPV4(+) and control groups. In contrast, there were no statistically significant alterations in pro-inflammatory genes (IL-1β, COX2, MMP3, and TNF-α). As Figure 5 shows, CD206 was significantly upregulated in the TRPV4(−) group, with an 8.99 ± 0.45-fold increase compared to control, and IL-10 in the TRPV4(−) group was upregulated, with 1.93 ± 0.6-fold compared to the control group which is statistically higher than relative fold change of IL-10 in TRPV4(+) group. However, the relative fold change of IL-1β, COX2, MMP3, and TNF-α increased with 1.41 ± 0.62-fold, 1.2 ± 0.62-fold, 1.46 ± 0.61-fold, and 2.42 ± 0.57-fold, respectively, compared with the control group but these changes were not statistically significant.

The heatmap in Figure 5 summarizes how pro- and anti-inflammatory gene expressions change as M1 macrophages are treated with TRPV4 modulators. The color gradients serve as a representation of the variations in z-scores corresponding to the relative fold change. The color transition from blue (negative z-score) to yellow (positive z-score) indicates gene upregulation. Overall, the gene expression data suggested that the TRPV4(+) group expressed dominantly pro-inflammatory markers and tended to preserve its pro-inflammatory phenotype. However, in the TRPV4(−) group, the anti-inflammatory markers were upregulated significantly which promoted phenotypic shifts of M1 macrophages from pro-inflammatory to pro-healing lineage.

### 3.5. Pharmacological Activation/Blockage of TRPV4 Channel in M1 Macrophages Promotes Pro-Inflammatory/Anti-Inflammatory Surface Protein Markers in M1 Macrophages

**Figure 6:**
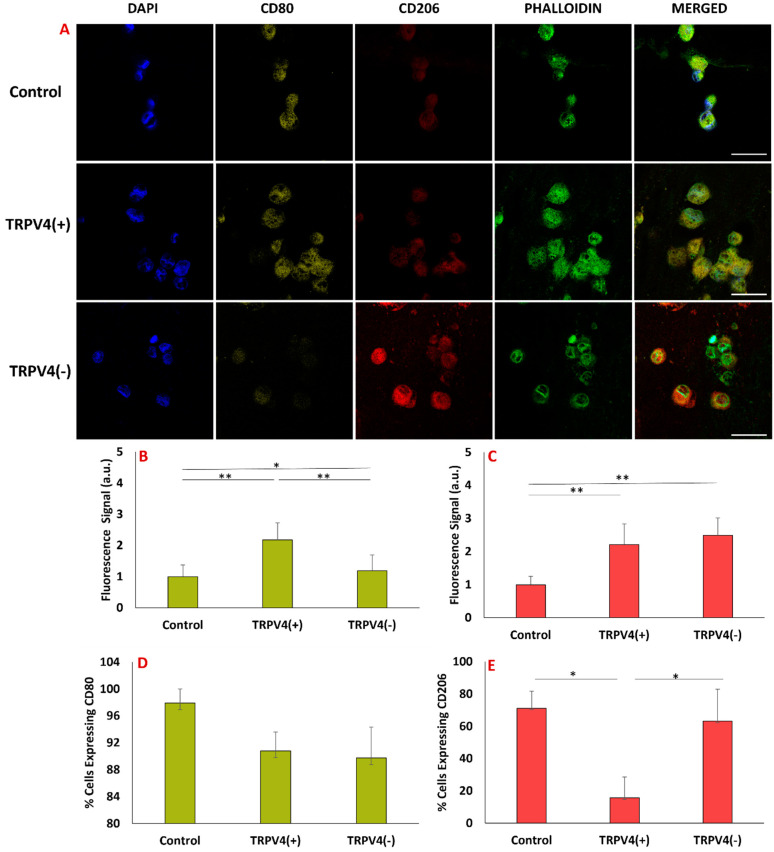
(**A**) Immunohistochemical staining for cell nucleus with DAPI, M1 marker CD80, M2 marker CD206, and F-actin with phalloidin. Representative of 5 images/group (scale bar = 25 μm). (**B**) The quantification of fluorescent intensity of CD80 in each group. (**C**) The quantification of fluorescent intensity of CD206 in each group. (**D**) The quantification of the percentage of cells expressing the CD80 marker in each group. (**E**) The quantification of the percentage of cells expressing the CD206 marker in each group. N = 60 cells were quantified for image analysis in each group; * and ** show the statistical difference between the groups with *p* < 0.05 and *p* < 0.005, respectively.

The phenotypic changes of TRPV4(+), TRPV4(−), and the control group were visualized and assessed using pro- and anti-inflammatory macrophage markers. The cell nuclei, filamentous actin (F-actin), pro-inflammatory surface markers, and anti-inflammatory surface markers were stained using DAPI, phalloidin, CD80, and CD206 antibodies, respectively (Figure 6A). Figure 6B shows that the expression of pro-inflammatory CD80 is more in the TRPV4(+) group compared to the TRPV4(−) and control groups. Figure 6C shows that the expression of anti-inflammatory CD206 is more in the TRPV4(−) group compared to TRPV4(+) and control groups.

Figure 6B demonstrates that the relative fluorescence intensity unit (RFU) of CD80 in the TRPV4(+) group is 2.17 ± 0.54 which is significantly higher than TRPV4(−) (1.19 ± 0.50) and the control group (1). Figure 6C shows a higher relative fluorescence intensity of CD206 in TRPV4(−) macrophages (2.48 ± 0.97) compared to TRPV4(+) (2.21 ± 0.76) and control group (1). Figure 6B,C collectively show that within the TRPV4(−) group, the expression of CD80 is lower compared to the other groups, while the expression of CD206 is higher compared to the other groups.

Figure 6D,E demonstrate the percentage of cells expressing CD80 marker and CD206 marker in each group, respectively. Percentages of cells expressing CD80 in control, TRPV4(+), and TRPV4(−) groups are 97.93 ± 2.06, 90.81 ± 2.77, and 89.76 ± 4.60, respectively. Percentages of cells expressing CD206 in control, TRPV4(+), and TRPV4(−) groups are 71.17 ± 10.48, 15.78 ± 12.80, and 63.13 ± 19.82, respectively.

## 4. Discussion

Modulating the TRPV4 channel has emerged as a potential therapeutic target to regulate inflammatory response [5]. Yet, considering the importance of the macrophage and its phenotypic changes in inflammatory response, not many studies have investigated the role of TRPV4 modulators on macrophage lineage commitment. There is a great demand to understand to what extent TRPV4 antagonists and agonists create phenotypic changes in pro-inflammatory macrophages (M1) and their surrounding three-dimensional (3D) matrix. Toward this end, the objective of this study was to investigate phenotypic changes in M1 macrophages within a 3D collagen matrix upon TPRV4 compound modulator treatments using comprehensive phenotypic and structural analyses. There are several prominent studies investigating the effectiveness of TRPV4 antagonists and agonists including RN-1734 and GSK1016790A on various cell types such as astrocytes, microglia, urothelial, endothelial, epithelial, chondrocytes, and murine macrophages through visualizing the TRPV4 channels with TRPV4-specific antibodies, monitoring the changes in intracellular Ca^2+^ influx, and running gene expressions on the cells with specific markers [42,43,44,45,46,47,48]. However, no studies have been conducted to understand and prove the effect of specific TRPV4 antagonists and agonists on human monocyte-derived macrophage differentiation using holistic and multiscale approaches incorporating differentiation surface marker assessment or cell-surrounding matrix interaction along gene expression analysis.

After validating the effectiveness of the TRPV4 modulator on macrophages at both the gene and protein levels and identifying the optimal TRPV4 antagonist concentration (Figure 2), a detailed structural analysis in cellular and collagen matrix levels was conducted upon TRPV4 antagonist and agonist treatment. The structural changes within the 3D collagen matrices upon TRPV4 modulator treatment were assessed using Masson’s trichrome and B-CHP staining (Figure 3). The assessment of collagen structural integrity circulating the M1 macrophages is important but largely ignored in TRPV4 modulator studies. The effect of TRPV4 antagonist and agonist treatments are solely characterized at the cellular level without considering the matrix where the cells are residing. However, several studies have demonstrated that increased inflammation markers affect the surrounding extracellular matrix of macrophages and remodel the tissue microenvironment [10,49]. This aspect of characterization is usually not incorporated because most of the studies conducted with TRPV4 compound modulators are performed with cell monolayers cultured in a Petri dish or well-plate, not with encapsulated cells within the 3D matrix. Masson’s trichrome staining of the TRPV4(+) group proved that pharmacological activation of TRPV4 channels in M1 macrophages resulted in less collagen content in the matrix (Figure 3A). To quantitively assess degraded collagen at the molecular level and specifically distinguish between intact and degraded collagen, matrices were stained with B-CHP. For the first time, this current study utilized the B-CHP staining method to study collagen degradation in macrophages treated with TRPV4 modulators. In 3D matrices, encapsulating TRPV4(+) macrophages, the lowest collagen content and highest proteolyzed collagen were observed due to inflammation in the matrix (Figure 3) [50,51]. Moreover, Figure 3 shows a correlation between loose extracellular matrix surrounding TRPV4(+) macrophages and the upregulation of MMP3 marker within the macrophages. This observation aligns with both existing literature and our prior research [28], illustrating that TRPV4 activation in cells promotes inflammation by enhancing MMP expression while inhibiting MMP inhibitor expression [49,50,51,52]. Overall, Figure 3 demonstrated the lower collagen content with the highest degradation in the macrophage-laden 3D matrix treated with the TRPV4 agonist (TRPV4(+)) group.

Once we had established the structural changes of the collagen matrix upon TRPV4 modulator treatment, we studied the structural changes in macrophages within the collagen matrix (Figure 4A). Previous studies have demonstrated that TRPV4 regulates cell morphology in a cell type-specific manner by forming molecular complexes containing cytoskeletal proteins and regulatory kinases [53,54]. For instance, Bagnell et al. [54] demonstrated that in neurological disease, TRPV4 activation results in the outgrowth of actin filaments through the activation of small GTPase RhoA. Similarly, Phoung et al. [55] showed that treating human retinal MVE cells with GSK1016790A disrupted the organization of F-actin and downregulated expression of occludin and remodeling of adherens junctions [55]. This current research investigated the effect of TRPV4 modulators on the cytoskeletal structures of the M1 macrophages encapsulated within the 3D matrix. Our study demonstrates that treating M1 macrophages encapsulated within a 3D matrix with TRPV4 agonist leads to the reorganization of filamentous actin (F-actin) into more spread and more elongated morphologies (Figure 4A). Also, as Figure 4B demonstrates in the TRPV4(−) group, cell surface area is less compared to TRPV4(+) and control groups. The smaller macrophage surface area of the TRPV4(−) group indicates that the macrophage phenotype has shifted toward an anti-inflammatory state. Pelegrin et al. [40] examined macrophage morphology across a polarity gradient, spanning from M1 to M2 phenotypes, and provided evidence of reduced cell area as macrophage polarity transitions from M1 to M2 [40]. Therefore, Figure 4 shows that M1 macrophage morphology can be altered upon TRPV4 channel treatment despite being embedded in the 3D collagen matrix. This finding is in contrast to the study of Lee et al. [56], that showed morphology of MSCs depends on 3D hydrogel in which they are embedded and did not change when treated with GSK1016790A [56]. Additionally, treating M1 macrophages with a TRPV4 antagonist reorganizes their cytoskeleton, resulting in a smaller surface area, indicative of a shift in phenotypic states toward an anti-inflammatory profile. Figure 3 and Figure 4 collectively demonstrate that alterations of 3D collagen structure through TRPV4 treatment can induce corresponding changes in the cytoskeletal structure of embedded macrophages.

Once the impact of TRPV4 channel modulation on M1 macrophage extracellular matrices and cytoskeletal structure was established, gene expression analysis was conducted (Figure 5). Prior studies on TRPV4 pharmacological modulation in macrophages mainly focused on characterizing the cytokine secretion level within the supernatant [57,58]; however, our research delves deeper into assessing the changes in the chemokine and cytokine levels using gene expression analysis. In the TRPV4(+) group, IL-1β, COX2, and TNF-α were significantly (*p* < 0.05) upregulated compared to the control group, which shows selective targeting of TRPV4 channel using TRPV4 agonist enhances inflammatory responses, as evidenced by upregulation of inflammatory markers. In the TRPV4(−) group, CD206 was statistically significantly (*p* < 0.005) upregulated compared to TRPV4(+) and control groups, and IL-10 was significantly upregulated compared to TRPV4(+) group. Overall, this data suggest that selectively blocking the TRPV4 channel in proinflammatory macrophages holds promise for controlling inflammation and promoting musculoskeletal (MSK) tissue regeneration.

To investigate whether the gene expression changes resulting from TRPV4 pharmacological modulation led to alterations in surface marker expression, we evaluated IHC images of TRPV4(+), TRPV4(−), and the control group. Figure 6 illustrates the phenotypic changes in M1 macrophages within a 3D matrix treated with TRPV4 modulators, as assessed by immunohistochemistry (IHC) for CD80 (pro-inflammatory marker) and CD206 (anti-inflammatory marker). Higher intensity of the CD80 fluorescent signal in the TRPV4(+) group compared to the TRPV4(−) and control groups demonstrates that pharmacological activation of the TRPV4 channel enhances pro-inflammatory properties of M1 macrophages. However, higher intensity of CD206 fluorescent signal in TRPV4(−) M1 macrophages compared to TRPV4(+) and control group demonstrates that pharmacological blockage of TRPV4 channel shifts M1 macrophages to the anti-inflammatory side of the macrophage phenotype spectrum which perfectly aligns with our gene-expression results. Figure 5 and Figure 6 together show that the treatment of M1 macrophages with a TRPV4 agonist and antagonist results in the polarization of macrophages toward pro-inflammatory and anti-inflammatory states, as substantiated by changes in both gene expression and surface protein levels.

This study aimed to investigate the impact of TRPV4 modulators on M1 macrophages encapsulated within collagen matrices, replicating the spatial constraints observed in musculoskeletal (MSK) tissue. By doing so, the study sought to explore the selective targeting of the TRPV4 channel and its influence on cell–cell and cell–matrix interactions, as well as the diffusion of signaling molecules. Understanding how TRPV4 channel modulators can induce a shift in macrophage phenotype toward a favorable state promises potential therapeutic applications.

## 5. Conclusions

Considering the pivotal role of macrophages in the inflammatory process, there is a need to elucidate TRPV4 modulation capacities in directing macrophage phenotypic shifts to control the inflammatory response. This study for the first time investigated the influence of TRPV4 channel modulation on M1 macrophages within 3D collagen matrices. The results indicate that inhibition of the TRPV4 channel in proinflammatory macrophages lessens their inflammatory characteristics, as evidenced by the decreased expression of pro-inflammatory genes and surface markers, along with the increased expression of anti-inflammatory genes and surface markers. Moreover, the phenotypic changes in M1 macrophages treated with TRPV4 modulators coincide with the structural changes in the microenvironment surrounding the M1 macrophages. Such findings hold significant promise for innovative approaches in musculoskeletal tissue regeneration and the precise control of inflammatory responses, opening new avenues for therapeutic interventions in regenerative medicine and immunology.

## Data Availability

The data are contained within the doscument otherwise can be shared upon request to the corresponding author.

## Supplementary Materials

The following supporting information can be downloaded at: https://www.mdpi.com/article/10.3390/biomedicines12010230/s1. Table S1. Forward and reverse primers for quantitative real-time polymerase chain reaction. References [34,59,60,61,62] are cited in Supplementary Materials.

## Abbreviations

CCL-18	Chemokine (C-C motif) ligand-18
CD163	Cluster of differentiation 163
CD206	Cluster of differentiation 206
COX-2	Cyclooxygenase-2
ECM	Extracellular matrix
FBS	Fetal bovine serum
GAPDH	Glyceraldehyde-3-phosphate dehydrogenase
IL	Interleukin
IL-10	Interleukin-10
LPS	Lipid polysaccharide
M0	Naïve macrophage
M1	Pro-inflammatory macrophage
M2	Anti-inflammatory macrophage
MMP	Matrix metalloproteinase
PBS	Phosphate buffered saline
PCR	Polymerase chain reaction
PMA	Phorbol 12-myristate 13-acetate
qRT-PCR	Quantitative real-time polymerase chain reaction
RFU	Relative fluorescence unit
RNA	Ribonucleic acid
RPMI	Roswell Park Memorial Institute
TNF-α	Tumor necrosis factor-α
U937	Human myeloid cell line
**List of Units and Symbols**	
M	Molar
U	Unit
*v*/*v*	Volume per volume
*w*/*v*	Weight per volume
μ	Micro
