# Supplementary material for: Modulating TRPV4 Channel Activity in Pro-Inflammatory Macrophages within the 3D Tissue Analog
Source: Biomedicines. 2024 Jan 19;12(1):230. doi: 10.3390/biomedicines12010230 (PMC10813551; doi:10.3390/biomedicines12010230)
Supplement: Supplementary file 1 [file biomedicines-12-00230-s001.zip › biomedicines-2810676-supplementary.pdf]

Table 1. Forward and reverse primers for quantitative real-time polymerase chain reaction

Gene	Forward Primer	Reverse Primer	Ref
iNOS	5'-CATGGTCCGCAAGAGAGTGC-3'	5'-AACGTAGACCTTGGGTTTGCC-3'	(1)
COX2	5′-CGGTGTTGAGCAGTTTTCTCC-3′	5′-AAGTGCGATTGTACCCGGAC-3′	(2)
MMP3	CAGCCAACTGTGATCCTGCT	CTTCATATGCGGCATCCACG	(3)
TNF-α	5'-AGAGGGAAGAGTTCCCCAGGGAC-3'	5'-TGAGTCGGTCACCCTTCTCCAG-3'	(4)
IL-1β	5'-CCAGCTACGAATCTCGGACCACC-3'	5'TTAGGAAGACACAAATTGCATGGTGAAGTCAGT-3'	(5)
CD163	5'-TCTGTTGGCCATTTTCGTCG-3'	5'TGGTGGACTAAGTTCTCTCCTCTTGA-3'	(4)
CD206	5’-CTTTAACGTGGCACCAGGCG-3’	5’-GCCAACCGCTGTTGAAGCTC-3’	(2)
IL-10	5'-CCTGTGAAAACAAGAGCAAGGC-3'	5'-TCACTCATGGCTTTGTAGATGCC-3'	(4)
GAPDH	5’-AGAAGGCTGGGGCTCATTTG-3’	5’-AGGGGCCATCCACAGTCTTC-3’	(6)

References

1. Teow A. Mechanoresponsive Naïve Human Macrophage Polarization within a 3D Collagen Matrix: University of Toledo; 2021.
2. Shortridge C, Akbari Fakhrabadi E, Wuescher LM, Worth RG, Liberatore MW, Yildirim-Ayan E. Impact of Digestive Inflammatory Environment and Genipin Crosslinking on Immunomodulatory Capacity of Injectable Musculoskeletal Tissue Scaffold. International Journal of Molecular Sciences. 2021;22(3):1134.
3. Jacho D, Rabino A, Garcia-Mata R, Yildirim-Ayan E. Mechanoresponsive regulation of fibroblast-to-myofibroblast transition in three-dimensional tissue analogues: mechanical strain amplitude dependency of fibrosis. Scientific Reports. 2022;12(1):16832.
4. Shortridge C, Akbari Fakhrabadi E, Wuescher LM, Worth RG, Liberatore MW, Yildirim-Ayan EJIJoMS. Impact of Digestive Inflammatory Environment and Genipin Crosslinking on Immunomodulatory Capacity of Injectable Musculoskeletal Tissue Scaffold. 2021;22(3):1134.
5. Li HX, Sze SCW, Tong Y, Ng TB. Production of Th1- and Th2-dependent cytokines induced by the Chinese medicine herb, Rhodiola algida, on human peripheral blood monocytes. Journal of Ethnopharmacology. 2009;123(2):257-66.
6. Shortridge C, Akbari Fakhrabadi E, Wuescher LM, Worth RG, Liberatore MW, Yildirim-Ayan E. Impact of Digestive Inflammatory Environment and Genipin Crosslinking on Immunomodulatory Capacity of Injectable Musculoskeletal Tissue Scaffold. Int J Mol Sci. 2021;22(3).
